# A case report of QT prolongation with glycopyrronium bromide in a patient with chronic tamoxifen use

**DOI:** 10.1186/s13104-016-2105-4

**Published:** 2016-06-14

**Authors:** Michael H. Chiu, Nawaf S. Al-Majed, Ryan Stubbins, Dylan Pollmann, Roopinder K. Sandhu

**Affiliations:** Division of Cardiology, Libin Cardiovascular Institute, University of Calgary, Calgary, AB Canada; Division of Cardiology, University of Alberta, Edmonton, AB Canada; Department of Internal Medicine, Faculty of Medicine, University of Alberta, Edmonton, AB Canada; Department of Pharmacy, Mazankowski Alberta Heart Institute, University of Alberta, Edmonton, AB Canada

**Keywords:** Chronic obstructive pulmonary disease, QT prolongation, Glycopyrronium bromide (Seebri), NVA237

## Abstract

**Background:**

Glycopyrronium bromide has recently been approved as a once daily maintenance inhalation therapy for moderate to severe chronic obstructive pulmonary disease (COPD). Efficacy and safety trial data have found rare cases of significant QT prolongation. To our knowledge, we describe the first case report of QT prolongation >600 ms with initiation of glycopyrronium bromide in a real world setting.

**Case presentation:**

A 78-year-old female with moderate COPD recently started on glycopyrronium bromide, presented to Emergency Department (ED) with syncope. Her past medical history was significant for a left total mastectomy and she had been on Tamoxifen for 9 months. One day prior to her presentation, she had visited a naturopathic clinic for a vitamin infusion resulting in emesis. The following day she continued to feel dizzy and had a witnessed syncopal episode without any reported cardiac or neurological symptoms preceding the event or after regaining consciousness. In the emergency department, she reported dizziness and was found to be hypotensive. Her symptoms completely resolved with intravenous fluids. Lab work was normal however her electrocardiogram (ECG) demonstrated a QTc interval of 603 and 631 ms (Friderica and Bazett’s respectively) with a normal QT interval on her baseline ECG prior to initiating Tamoxifen. She was admitted to the Cardiology service for further work-up of QT prolongation. Her syncope was felt to be due to orthostatic hypotension and the QT prolongation secondary to medications, which were both discontinued during her admission. After 2 days, her QT interval normalized consistent with the half-life of Glycopyrronium bromide (13–57 h) compared to Tamoxifen (8–14 days).

**Conclusion:**

Glycopyrronium bromide is guideline recommended as first line therapy for prevention of exacerbation in moderate to severe COPD however safety data had been limited to select populations. This case report highlights the need for future studies to identify high-risk populations at potential risk of life-threatening arrhythmias who may benefit from periodic ECG surveillance.

## Background

Chronic obstructive pulmonary disease (COPD) is a common condition, comprising of a spectrum of emphysema, bronchitis and bronchiolitis that involves parenchymal destruction and infiltration [1]. Pharmacologic maintenance therapy for COPD involves bronchodilation via β-2 adrenergic agonist, corticosteroids, anticholinergics and long acting muscarinic antagonists (LAMA) [2]. Glycopyrronium bromide (GB) is a recently approved synthetic competitive muscarinic antagonist that acts at the bronchial smooth muscle and inhibits acetylcholine-mediated bronchoconstriction. GB is recommended as first line therapy for preventing exacerbations in moderate to severe COPD [3].

Efficacy and safety trial data in selected populations have found variable results regarding QT prolonging effects with GB therapy and rare cases of QT prolongation >500 ms [4–9]. A small, randomized controlled study found no repolarization abnormalities with a single supra-therapeutic dosing of GB in young, healthy adults [10]. After extensive investigation and consideration of other possible offending medication, we report here the first case of severe and transient QT prolongation consistent with pharmacokinetics of GB in the real-world setting.

## Case presentation

A 78-year-old female with a past medical history significant for breast cancer and moderate COPD presented to Emergency Department with syncope. Nine months prior to admission, she underwent left total mastectomy for invasive ductal carcinoma and was started on Tamoxifen 20 mg daily. Home medications included GB 50 mcg once daily, Salbutamol, calcium and a multi-vitamin. GB was started 3 months prior to her presentation for syncope.

**Fig. 1:**
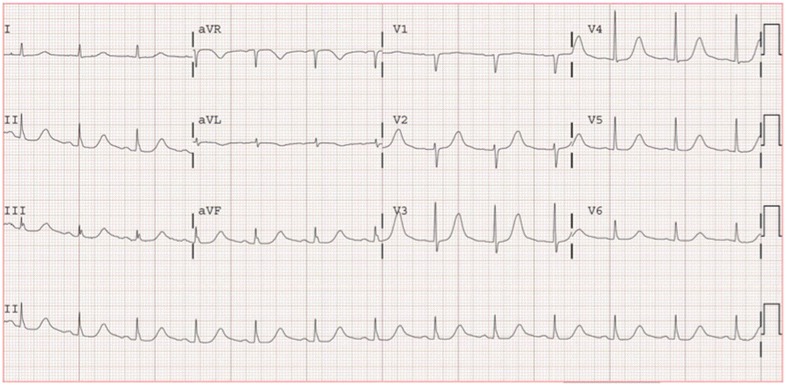
Electrocardiogram at hospital presentation demonstrating prolonged corrected QT intervals of 603 ms using Fridericia’s equation and 631 ms using Bazett’s formula

The day prior to admission, she received an IV vitamin infusion consisting of a mixture of thiamine, folic acid, multivitamin and magnesium sulfate at a naturopath clinic. Shortly after completion of the IV infusion, she developed emesis and took 2 tablets of dimenhydrinate. The following day, she reported dizziness as she walked across the kitchen and passed out after sitting in a chair. The patient reported no palpitations, chest discomfort, nausea, warm sensation or diaphoresis prior to the syncope event. She was not witnessed to have any seizure like activity and when she regained consciousness, reported immediate awareness of surroundings with no neurological deficits, no tongue biting, bowel or urinary incontinence. There was no previous history of syncope and no family history of sudden cardiac death. Her initial blood pressure in the ambulance was 70/50 mm Hg. Hemodynamics normalized after administration of intravenous fluid and her symptoms resolved. Oxygen saturation was above 97 % and telemetry revealed normal sinus rhythm with a heart rate of 77. Precordial examination was unremarkable with regular normal heart sounds and no murmurs. In the Emergency Department, blood tests including complete blood count (CBC), serum electrolytes (potassium, calcium, magnesium), glucose, creatinine and thyroid stimulating hormone were normal. Electrocardiogram (ECG) showed a corrected QT interval using Fridericia (QTcF) and Bazett’s formula (QTcB) of 603 and 631 ms respectively (Fig. 1) and she was admitted to the cardiology service for further investigation of the etiology for her QT prolongation.

**Fig. 2:**
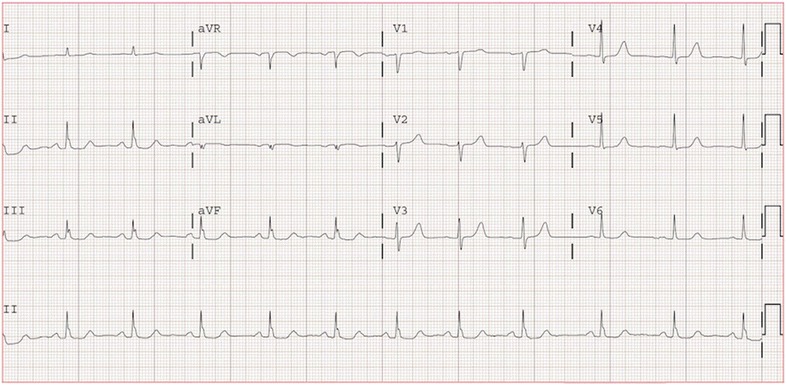
Electrocardiogram prior to starting Tamoxifen with corrected QT intervals of 439 ms using Fridericia’s equation and 440 ms using Bazett’s formula

Prior to starting Tamoxifen her QTcF and QTcB were 439 and 440 ms respectively (Fig. 2), however no ECG was obtained after initiation of Tamoxifen and prior to starting GB. Her last dose of Tamoxifen and GB were the day of admission with both medications discontinued at presentation.

**Fig. 3:**
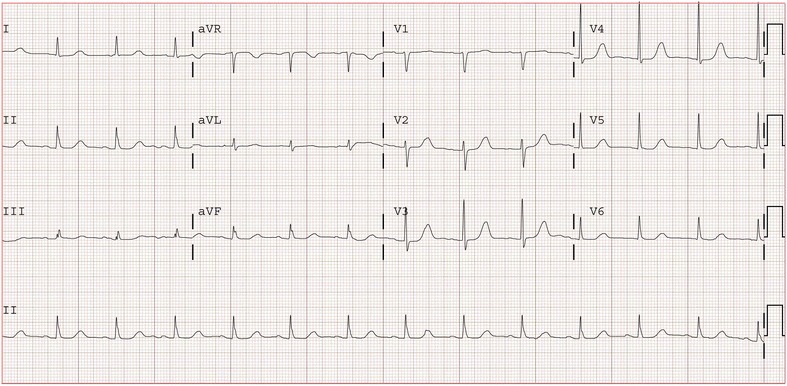
Electrocardiogram 72 h after stopping glycopyrronium with corrected QT intervals of 442 ms using Fridericia’s equation and 460 ms using Bazett’s formula

An echocardiogram revealed that her left ventricular ejection fraction was >60 % with no valvular or regional wall motion abnormalities. Serial electrocardiograms demonstrated corrected QTcF and QTcB respectively of 603 and 631 ms day 0, 496 and 514 ms day 1, and 446 and 455 ms on day 2 and 442 and 460 ms on day 3 (Fig. 3). There were no arrhythmias seen on telemetry. Syncope was felt to be secondary to orthostatic changes and she was discharged on day 3 after admission.

## Discussion

The QT interval represents the time interval between the onset of the QRS complex and the end of the T wave including ventricular depolarization and repolarization. There are many factors that can induce QT prolongation including left ventricular hypertrophy, heart failure, myocardial ischemia, hypertension, diabetes, thyroid dysfunction, bradycardia and electrolyte abnormalities including hypokalemia and hypomagnesaemia. Congenital long QT syndrome (LQTS) is due to direct mutations in specific genes such as the cardiac delayed rectifier potassium channel (KCNQ1 and KCNH2) or the cardiac sodium channel (SCN5A). In congenital LQTS, exercise, emotional or physical stress is presumed to be an underlying trigger for long QT events. Aside from congenital LQTS, there is a 30 % heritability of the QT interval in the general population [11].

Medications represent one of the largest causes of prolonged QT [12]. The primary mechanism of drug induced QT prolongation is blockade of the rapid component of the delayed rectifier potassium channel leading to prolonged action potential duration [13]. Drugs that inhibit the human ether-à-go-go-related gene (hERG) K+ channel have the highest risk of QT prolongation. HERG inhibition increasing susceptibility to early after depolarization that can result in torsades de pointes (TdP) and a risk of sudden cardiac death [11, 12, 14].

In our case, the possible offending drugs responsible for QT prolongation were GB and Tamoxifen. The pharmacokinetics of the two drugs differs considerably; the half-life of GB is 13–57 h while that of Tamoxifen is 4–9 days with a single dose. Clinical studies of QT prolongation associated with Tamoxifen reported normalization of the QT interval within 8–14 days after drug cessation [15, 16]. There are no known drug interactions between the anticholinergic properties of GB and estrogen receptor properties of Tamoxifen and both have different excretion pathways [17]. The mechanism of action for GB is via Muscarinic M1 and M3 receptor blockade, which prevents parasympathetic mediated bronchoconstriction [18]. GB has less selectivity toward the M2 receptor present in the heart, pacemaker cells and conduction system [18].

In experimental studies, the effects on the hERG K^+^ channel blockade channels have previously been examined during product safety development. In-vitro patch clamp experiments reported a progressive dose dependent inhibition of hERG current [19]. Inhibition of the hERG current was 0.6 % for the vehicle and 13.2, 18.3 and 30.6 % for 3.2, 31.8 and 95.5 μg/mL of GB respectively. Novartis product normogram mentions an IC50 value of >300μM, which is greater than 500,000 times of the maximal recommended concentration C_max_ [19, 20]. Electrophysiological rabbit studies reported no proarrhythmic activity at supertherapeutic dosing. Furthermore, telelmetry studies on beagle dogs with supertherapeutic IV GB (12 > C_max_ in humans) reporting a transient QTc shortening of up to 30 s observed for up to 2 h [20].

In clinical studies, the safety and efficacy of GB have been studied in six randomized controlled studies (GLycopyrronium bromide in COPD airWays—GLOW) [4–9]. The safety profile testing for GB in the GLOW trials excluded patients with a QTc greater than 450 ms in males and 470 ms in females. Other exclusion criteria included moderate or severe renal dysfunction, prior lung cancer, active infection, urinary retention or history of alpha 1 anti-trypsin. A history of breast cancer was not specifically reported as an exclusion criterion. Three of these trials reported slightly variable results ranging from no significant incidence to low incidence of QT prolongation in GB compared to placebo or active comparator, and rare reports of QT prolongation >500 ms (Table 1) [4–9]. In the GLOW trials no significant QT prolongation was noted between GB versus placebo. The variable QT results in the GLOW trials prompted a randomized, partially blind, placebo and positive (moxifloxacin) control, 3 way cross-over study among 73 healthy adults [10]. Compared to placebo, no significant QT prolonging effect was observed after inhalation of a single supra-therapeutic (400 μg) dose of GB.

**Table 1:** Summary Table of the glycopyrronium QT_CF_ effects in the GLOW 1–6 trials (the GB in COPD airWays studies)[4–9]

Aim of study; study size (N)	Patient population	Study intervention Study comparator	QT prolongation
GLOW-1 Aim Efficacy, safety and tolerability of GB in moderate/severe COPD Study type Randomized controlled trial double blind Size n = 822	*Inclusion criteria* GOLD criteria—moderate to severe ≥40 years of age Smoking history of ≥10 pack-years Exclusion criteriaPost-bronchodilator FEV_1_ of <80 and ≥30 % of predicted FEV_1_/FVC ratio of <0.70 Lower respiratory tract infection (RTI) within 6 weeks; concomitant pulmonary disease history of asthma or lung cancer Long QT syndrome: QTc >450 ms (males) or >470 (females) Symptomatic prostatic hyperplasia Bladder-neck obstruction Moderate/severe renal impairment Urinary retention Narrow—angle glaucoma History of alpha-1 antitrypsin	*Intervention* GB n = 552, completed 26 week trial n = 450 *Comparator* Placebo n = 270, completed 26 week trial n = 212	*QTcF >500 ms* GB: n = 0 (0 %) Placebo: n = 0 (0 %) * Increase of 30–60 ms* GB: n = 59 (10.7 %) Placebo n = 21 (7.9 %) *Increase of >60 ms* GB: n = 6 (1.1 %) Placebo: n = 1 (0.4 %)
GLOW-2 Aim Efficacy, safety and tolerability of GB in moderate/severe COPD vs placebo vs tiotropium Study type Randomized controlled trial (double blind) Size n = 1066	*Inclusion criteria* Males and females ≥40 years of age Smoking history of ≥10 pack-years GOLD moderate-to-severe COPD Post-bronchodilator FEV1 ≥30 and <80 % of the predicted Post-broncho-dilator FEV1/FVC <0.70 *Exclusion criteria* Lower RTI 6 weeks prior Concomitant pulmonary disease	*Intervention* GB n = 529, completed 52 week trial n = 411 *Comparator* Placebo n = 269, completed 52 week trial n = 193 Tiotropium n = 268 completed 52 week trial n = 206	*QTcF >500 ms* GB: n = 2 (0.4 %) Placebo: n = 2 (0.7 %) Tiotropium: n = 0 (0 %) *Increase of 30–60 ms:* GB: n = 83 (15.8 %) Placebo: n = 39 (14.6 %) Tiotropium: n = 43 (16.2 %) *Increase of >60 ms* GB: n = 1 (0.2 %) Placebo: n = 1 (0.4 %) Tiotropium: n = 0 (0 %)
	Pulmonary tuberculosis		
	Bronchiectasis		
	History of asthma		
	Malignancy of any system		
	Long QT syndrome or QTc >450 ms (males) or >470 (females) Symptomatic prostatic hyperplasia Bladder-neck obstruction Moderate/severe renal impairment Urinary retention Narrow-angle glaucoma History of a_1_-antitrypsin deficiency Active in pulmonary rehabilitation Contraindications for tiotropium or ipratropium or history of adverse reactions to inhaled anticholinergics		
GLOW-3 Aim Exercise tolerance with once daily GB Study type Randomized Controlled Trial (Double blind) Size n = 108	*Inclusion criteria* GOLD moderate to severe COPD Aged ≥ 40 years Smoking history ≥10 pack-years Post-bronchodilator FEV_1_ <80 % ≥40 % of predicted normal Post-bronchodilator FEV_1_/FVC of <70 %. *Exclusion criteria* Lower RTI prior 6 weeks Oxygen for chronic hypoxemia Concomitant pulmonary disease	*Intervention* GB n = 55 *Comparator* Placebo n = 53	QTcF—not measured
	Pulmonary tuberculosis		
	Bronchiectasis		
	History of asthma		
	Malignancy of any system		
	Long QT syndrome QTc >450 ms for males or >470 ms for females, Symptomatic prostatic hyperplasia Bladder-neck obstruction Moderate/severe renal impairment Urinary retention Narrow-angle glaucoma Alpha-1 anti- trypsin deficiency Adverse reactions to inhaled anticholinergic agents, long- and short-acting alpha-agonists or Sympathomimetic amines Active pulmonary rehabilitation		
GLOW-4 Aim Efficacy, safety and tolerability of GB in moderate/severe COPD vs tiotropium in the Japanese Population Study type Randomized controlled trial (Double blind) Size n = 163	*Inclusion criteria* GOLD moderate-to-severe COPD Males and females ≥40 years of age Smoking history of ≥10 pack-years GOLD moderate-to-severe COPD Post-bronchodilator FEV1 ≥30 and <80 % of the predicted Post-broncho-dilator FEV1/FVC <0.70 *Exclusion criteria* Lower RTI prior 6 weeks Oxygen for chronic hypoxemia Concomitant pulmonary disease	*Intervention* GB n = 123 *Comparator* Placebo n = 40	QTcF—not measured
	Pulmonary tuberculosis		
	Bronchiectasis		
	History of asthma		
	Malignancy of any system		
	Long QT syndrome QTc >450 ms for males or >470 ms for females, Symptomatic prostatic hyperplasia Bladder-neck obstruction Moderate/severe renal impairment Urinary retention Narrow-angle glaucoma Alpha-1 anti- trypsin deficiency Adverse reactions to inhaled anticholinergic agents, long- and short-acting alpha-agonists or sympathomimetic amines Active pulmonary rehabilitation		
GLOW-5 Aim Efficacy, safety and tolerability of GB in moderate/severe COPD vs once daily tiotropium Study type Randomized controlled trial (double blind) Size n = 657	*Inclusion criteria* GOLD moderate-to-severe COPD Males and females ≥40 yrs of age Smoking history of ≥10 pack-yrs GOLD moderate-to-severe COPD Post-bronchodilator FEV1 ≥30 % and <80 % of the predicted Post-broncho-dilator FEV1/FVC <0.70 *Exclusion criteria* Lower RTI prior 6 weeks Oxygen for chronic hypoxemia Concomitant pulmonary disease	*Intervention* GB n = 327, completed 12 week trial n = 314 *Comparator* Tiotropium n = 320, completed 12 week trial n = 316	*QTcF >480 ms* GB: n = 2 (0.64) % Tiotropium: n = 0 (0 %) *QTcF increase 30–60 ms:* GB: n = 11 (3.4 %) Tiotropium: n = 10 (3 %)
	Pulmonary tuberculosis		
	Bronchiectasis		
	History of asthma		
	Malignancy of any system		
	Long QT syndrome QTc > 450 ms for males or > 470 ms for females, Symptomatic prostatic hyperplasia Bladder-neck obstruction Moderate/severe renal impairment Urinary retention Narrow-angle glaucoma Alpha-1 anti- trypsin deficiency Adverse reactions to inhaled anticholinergic agents, long- and short-acting alpha-agonists or sympathomimetic amines Active pulmonary rehabilitation		
GLOW-6 Aim Efficacy, safety and tolerability of GB and indacaterol vs indacaterol alone in moderate/severe COPD Study type Randomized controlled trial (double blind) Size n = 449	*Inclusion criteria* GOLD moderate-to-severe COPD Males and females ≥ 40 yrs of age Smoking history of ≥ 10 pack-yrs GOLD moderate-to-severe COPD Post-bronchodilator FEV1 ≥ 30 % and < 80 % of the predicted Post-broncho-dilator FEV1/FVC < 0.70 *Exclusion criteria* Lower RTI prior 6 weeks Oxygen for chronic hypoxemia Concomitant pulmonary disease	*Intervention* GB + Indacaterol n = 226 Completed 12 week trial n = 212 *Comparator* Indacaterol n = 223 completed 12 week trial n = 210	*QTcF >500 ms:* GB +Ind: n = 0 (0 %) Ind: n = 0 (0 %) *QTcF increase 30–60 ms:* GB + Ind: n = 14 (6.5 %) Ind: n = 9 (4.2 %)
	Pulmonary tuberculosis		
	Bronchiectasis		
	History of asthma		
	Malignancy of any system		
	Long QT syndrome QTc > 450 ms for males or > 470 ms for females, Symptomatic prostatic hyperplasia Bladder-neck obstruction Moderate/severe renal impairment Urinary retention Narrow-angle glaucoma Alpha-1 anti- trypsin deficiency Adverse reactions to inhaled anticholinergic agents, long- and short-acting alpha-agonists or sympathomimetic amines Active pulmonary rehabilitation Atrial fibrillation NYHA III or IV symptoms		

*FEV*_*1*_ forced expiratory volume in 1 second, *FVC* forced vital capacity, *RTI* respiratory tract infection

## Conclusion

Glycopyrronium bromide is guideline recommended as first line therapy for prevention of exacerbation in moderate to severe COPD however safety data particularly in regards to QT prolongation has been limited to select populations. In our case, we demonstrate severe QT prolongation associated with Glycopyrronium bromide, emphasizing the need for further studies in a real-world setting to identify high-risk patients that may benefit from ECG surveillance.

## Abbreviations

COPD: chronic obstructive pulmonary disease
ED: Emergency Department
ECG: electrocardiogram
LAMA: long acting muscarinic antagonists
GB: glycopyrronium bromide
CBC: complete blood count
QTcF: corrected QT interval using Fridericia
QTcB: corrected QT interval using Bazett’s formula
LQTS: long QT syndrome
TdP: torsades de pointes
GLOW: gLycopyrronium bromide in COPD airways
